# Spatial Distribution of Macrophages During Callus Formation and Maturation Reveals Close Crosstalk Between Macrophages and Newly Forming Vessels

**DOI:** 10.3389/fimmu.2019.02588

**Published:** 2019-11-26

**Authors:** Jonathan Stefanowski, Annemarie Lang, Ariana Rauch, Linus Aulich, Markus Köhler, Alexander F. Fiedler, Frank Buttgereit, Katharina Schmidt-Bleek, Georg N. Duda, Timo Gaber, Raluca A. Niesner, Anja E. Hauser

**Affiliations:** ^1^Department of Rheumatology and Clinical Immunology, Charité—Universitätsmedizin Berlin, Corporate Member of Freie Universität Berlin, Humboldt-Universität zu Berlin, and Berlin Institute of Health, Berlin, Germany; ^2^German Rheumatism Research Centre (DRFZ) Berlin, a Leibniz Institute, Berlin, Germany; ^3^Berlin-Brandenburg Center for Regenerative Therapies, Charité—Universitätsmedizin Berlin, Corporate Member of Freie Universität Berlin, Humboldt-Universität zu Berlin, and Berlin Institute of Health, Berlin, Germany; ^4^Julius Wolff Institute for Biomechanics and Musculoskeletal Regeneration, Charité—Universitätsmedizin Berlin, Corporate Member of Freie Universität Berlin, Humboldt-Universität zu Berlin, and Berlin Institute of Health, Berlin, Germany; ^5^Dynamic and Functional in vivo Imaging, Department of Veterinary Medicine, Freie Universität Berlin, Berlin, Germany

**Keywords:** bone regeneration, macrophage, endothelial cell, H-type vessel, intravital microscopy, LIMB, CX3CR1 myeloid cells

## Abstract

Macrophages are essential players in the process of fracture healing, acting by remodeling of the extracellular matrix and enabling vascularization. Whilst activated macrophages of M1-like phenotype are present in the initial pro-inflammatory phase of hours to days of fracture healing, an anti-inflammatory M2-like macrophage phenotype is supposed to be crucial for the induction of downstream cascades of healing, especially the initiation of vascularization. In a mouse-osteotomy model, we provide a comprehensive characterization of vessel (CD31^+^, Emcn^+^) and macrophage phenotypes (F4/80, CD206, CD80, Mac-2) during the process of fracture healing. To this end, we phenotype the phases of vascular regeneration—the expansion phase (d1–d7 after injury) and the remodeling phase of the endothelial network, until tissue integrity is restored (d14–d21 after injury). Vessels which appear during the bone formation process resemble type H endothelium (CD31^hi^Emcn^hi^), and are closely connected to osteoprogenitors (Runx2^+^, Osx^+^) and F4/80^+^ macrophages. M1-like macrophages are present in the initial phase of vascularization until day 3 post osteotomy, but they are rare during later regeneration phases. M2-like macrophages localize mainly extramedullary, and CD206^+^ macrophages are found to express Mac-2^+^ during the expansion phase. VEGFA expression is initiated by CD80^+^ cells, including F4/80^+^ macrophages, until day 3, while subsequently osteoblasts and chondrocytes are main contributors to VEGFA production at the fracture site. Using Longitudinal Intravital Microendoscopy of the Bone (LIMB) we observe changes in the motility and organization of CX3CR1^+^ cells, which infiltrate the injury site after an osteotomy. A transient accumulation, resulting in spatial polarization of both, endothelial cells and macrophages, in regions distal to the fracture site, is evident. Immunofluorescence histology followed by histocytometric analysis reveals that F4/80^+^CX3CR1^+^ myeloid cells precede vascularization.

## Introduction

Bone healing is a spatiotemporally regulated regeneration process, ideally leading to complete restoration of the broken bone without fibrous scar formation (1). Naturally, this regeneration process undergoes endochondral bone formation, if interfragmentary movements strain the fracture gap (2). In the majority of clinical cases, fracture healing follows the endochondral route and may be sub-divided into five phases, namely an (i) initial pro-inflammatory phase, (ii) anti-inflammatory phase, (iii) fibrocartilaginous or soft callus phase, (iv) mineralization or hard callus phase and (v) remodeling phase in which bone tissue regains its physiological shape with a restored bone marrow cavity. While fracture healing occurs in most cases without delay, still 5–10% of patients suffer from delayed healing or non-union. To avoid delayed healing and overcome non-unions, it is important to understand the finely orchestrated processes of bone regeneration (3, 4).

Upon a fracture, the vessels get disrupted and nutrient supply is lacking at the injury site. However, the vascular system is essential for healing, by supplying cells with oxygen and nutrients, removing debris and allowing the recruitment of circulating cells. Endothelial progenitors (CD31^+^) migrate to the fracture site from the bone marrow or from pre-existing vessels of the periosteum (5–9). In earlier work, we could show that revascularization peaks during two phases of fracture healing: around day 7 (end of the inflammatory phase) and around day 21 (woven bone formation) in sheep (6). Drastic vascular structural plasticity has also been shown during bone marrow regeneration between 7 and 21 days by our group using a longitudinal microendoscopic method at cellular resolution (10). Angiogenic factors, such as vascular endothelial growth factor (VEGF) are of great importance in order to induce vascularization. Street et al. showed that the circulating plasma levels of VEGF are highly increased in patients with fractures and that the fibrin matrix within the fracture hematoma acts like a VEGF reservoir (11). In addition, we reported in a previous study that cells within the fracture hematoma exhibit upregulated VEGF expression and secretion (12, 13). Osteoprogenitor cells also produce VEGF as a consequence of the hypoxic environment, leading to enhanced transcriptional activity of hypoxia-inducible factor 1-alpha (HIF-1α) (14, 15). Buettmann et al. most recently showed that especially the release of VEGFA by Osterix (Osx)^+^ osteoprogenitors/pre-osteoblasts is critical for vessel formation during fracture healing (16). It is well-known that the crosstalk between endothelium and bone cells is essential for the integrity and formation of bone. Osteoblasts support the vasculature by producing VEGF and basic fibroblast growth factor (bFGF) while endothelial cells (ECs) provide factors that further osteoblast differentiation and activity (17). Furthermore, pre-osteoclasts and non-bone-resorbing osteoclasts have been described to enhance vascularization and osteogenesis in the growth plate area by releasing platelet-derived growth factor-BB (PDGF-BB) or supporting vessel anastomosis (18, 19). While the production of angiogenic factors by osteoprogenitors is well-known, there is evidence accumulating that, conversely, endothelial cells can also impact on bone formation, at least during bone development (7). Bone development is initiated by blood vessel invasion, and the arrival of osteoprogenitors. Subsequently, specialized type H blood vessels secrete osteogenic factors, required for the induction of bone formation and growth (20). Although the presence of type H vessels has been reported at sites of bone regeneration (10, 21), early events inducing the formation of these vessels in those situations have not yet been investigated.

Next to the adaptive immunity, the importance of the innate immune system for regenerative processes has been shown by several studies (22–26). Macrophages have been identified as key players for the recovery of tissue integrity and function. Several different types of myeloid-lineage cells can be distinguished in bone regeneration (23). Tissue-resident macrophages (also termed osteomacs) which express F4/80, can be found closely to bone-lining cells, and support intramembranous bone formation as well as endochondral ossification (22, 25, 26). Recruited immune macrophages (M1-like/M2-like) are more pivotal in endochondral ossification, which has been shown by Schlundt et al., who deleted macrophages in osteotomized mice by treatment with clodronate liposomes, and Alexander et al. who examined macrophage subsets close to the periosteum during regeneration (24, 25). Furthermore, osteoclasts are multinucleated tartrate-resistant acid phosphatase (TRAP)^+^F4/80^−^ myeloid cells, which derive from fusion events (22).

In other tissues or scenarios, macrophages are essential for vascularization and angiogenesis. They are able to degrade extracellular matrix (ECM) and release pro-angiogenic factors. Degradation of the ECM enables the migration of endothelial progenitors and activates the angiogenic potential of some ECM molecules, as has been shown for fragments of hyaluronic acid (27). In addition, macrophages also release factors that attract, activate or even inhibit angiogenic cells depending on the phase of vascularization (28). Studies during mouse development revealed the tight association of macrophages with capillaries and the subsequent enhancement of angiogenesis (29, 30). Macrophages have also been shown to regulate vessel permeability comparable to pericytes (31). *Vice versa*, endothelial cells (ECs) also promote the selective growth and differentiation of macrophages, especially the switch towards an M2-like phenotype, which requires direct contact with the endothelium and the regulation via macrophage colony-stimulating factor (M-CSF) signaling (32). However, a potential crosstalk between macrophages and ECs and the details of such interactions during bone regeneration have not been studied so far.

Within this study, we aim to analyze the initial phases of angiogenesis in bone healing with vascular regeneration and their dependence on the presence of macrophages. We focus on the early regeneration events, until the shift from pro- to anti-inflammatory phase, where a close crosstalk of blood vessels with macrophages is most prominent (d1–d7 after injury) and compare this to the bone remodeling phase driven by osteoprogenitors and mineralized tissue formation (d14–d21 after injury).

## Methods

### Animal Husbandry, Housing, and Surgery

#### Mouse-Osteotomy-Model

All animal experiments were approved by the local animal protection authority (LaGeSo; permit numbers: G0039/16 and G0111/13) following the German Animal Welfare Act.

Female C57BL/6N mice aged 10 weeks were ordered from Charles River Laboratories (Sulzfeld, Germany) and underwent surgery at the age of 12 weeks with an average body weight of 22 g. Housing took place in a conventional, semi-barrier (non-SPF) facility and randomly split in groups with at least 2 mice per cage housed in Eurostandard Type II clear-transparent plastic cages with a wire lid and filter top. Fine wood chips (Lignocel FS 14, J. Rettenmaier & Söhne GmbH + Co. KG, Germany) and nesting material (Envirodri^®^, Shepherd Specialty Papers, USA) was provided. Houses and pipes were removed after surgery to avoid injuries due to the external fixator. Food (Standard mouse diet, Ssniff Spezialdiäten, Germany) and tap water was provided *ad libitum*, and room temperature was between 20 and 22°C with a humidity of 45–50%. The light/dark cycle was a 12/12-h cycle. Animals were tail and cup handled. Anesthesia was induced at 2.5% isoflurane (CP-Pharma, Germany) and maintained at 1.5%. In order to cover pain after the surgery prior to surgery, all animals received Buprenorphine (0.03 mg/kg; Temgesic, Indivior Eu Ltd., UK) s.c. as analgesic, an eye ointment and clindamycin (0.02 ml; Ratiopharm, Germany). After shaving and disinfecting the left femur area animals were placed on a heating mat and osteotomy was performed under aseptic conditions as described earlier (21, 24). In short, the femur was prepared bluntly, after a lateral longitudinal incision of the skin between hip and knee. The external fixator (MouseExFix, RISystem, Switzerland) was placed parallel to the femur by serial drilling of the pins (0.45 mm diameter). With a Gigli wire saw (RISystem, Davos, Switzerland), a 0.70 mm osteotomy gap was created in the middle of the femur and flushed with NaCl. Following skin closure, mice received pre-warmed NaCl (0.2 ml) s.c., permeable wound dressing spray and could recover from anesthesia in their home cage under infrared light and close monitoring. Tramadol was applied via the drinking water (0.1 mg/ml; Grünenthal, Germany) for 3 days after osteotomy (33). Given the short time period of treatment, we expect no negative influence of the analgetics on the fracture healing outcome (33). Surgery was performed by two trained veterinarians. For general scoring and humane endpoints, optimized protocols were used which has been summarized in Lang et al. (34).

#### Combined Osteotomy and Intravital Imaging Model

All animal experiments were approved by the local animal protection authority (LaGeSo; permit numbers: G0302/17) following the German Animal Welfare Act.

Cx3cr1^tm1Litt^ (CX3CR1:GFP), a fractalkine receptor (CX3CR1) reporter mouse, and C57BL/6J animals were bred in our colony. Heterozygous female mice were 14 weeks of age when osteotomy was performed. Housing took place in a conventional SPF barrier facility. Prior to surgery, all animals received Buprenorphine (0.03 mg/kg; Temgesic, Indivior Eu Ltd., UK) s.c. as analgesic and eye ointment. After shaving and disinfecting the left femur area animals were placed on a heating mat and osteotomy was performed under aseptic conditions. Surgery was performed as previously described (10), using four bi-cortical screws, and combined with osteotomy. In short, the internal fixator's Gradient Refractive INdex (GRIN) lens tubing was modified to be screwed into the fixator plate after implantation and osteotomy. Osteotomy was performed using a 0.22 mm Gigli wire saw (RISystem, Switzerland) and two cuts for an osteotomy gap size of ~816 μm (CI: 787–844 μm; *SD* = 85 μm; *n* = 37). After removing the generated bone piece, the lens tube was positioned into the osteotomy gap and screwed into the fixator plate. Analgetics (Tramadol, Buprenorphine) were applied as described above. For antibiotic treatment mice received one injection of 0.04% Enrofloxacin (Baytril, 10 mg/kg body weight Bayer AG, Germany) before surgery.

### Bone Sample Preparation

Femoral bones were explanted, muscles largely removed in a way that osteotomized bone parts maintained one entity. Tissue was fixed using 4% electron microscopy-grade PFA in PBS for 4 h at 4°C, washed in PBS, and ran through a sucrose gradient (10%, 20%, 30%; á 12–24 h). The fixators were removed from the fixed samples, underwent μCT measurement, bones were frozen in SCEM medium (Sectionlab, Japan), cut into slices of 7 μm using Kawamoto‘s film method (35), and stored at −80°C.

### Histology

Movat's Pentachrome staining was conducted as descried previously (21, 24). TRAP staining for quantification was performed using a kit following the manufacturer's instructions (Thermo Scientific, 386A-1KT, MA, US). Individual slides were stained using small volumes of staining solutions on a heating plate at 37°C. For immunofluorescence, individual sections were thawed, rehydrated in PBS, blocked with 10% donkey serum, and stained with antibodies in PBS/0.1% Tween 20/5% donkey serum containing DAPI for 1–2 h. Target proteins were identified using antibodies against CD31/PECAM-1 (goat polyclonal unconjugated, AF2628, R&D Systems, 1:100), CD206/MMR (C068C2 conjugated to AF594, BLD-141726, 1:100), CD80 (goat polyclonal unconjugated, AF740-SP, 1:100), Endomucin (Emcn) (V.7C7 unconjugated, sc-65495, 1:100), F4/80 (Cl:A3-1 unconjugated, MCA497G, 1:400), GFP (goat polyclonal conjugated to AF488, 600-101-215, 1:100), Ly-6C (ER-MP20 biotinylated, MA5-16666, 1:20), Ly-6G (1A8 biotinylated, BLD-127603, 1:200), Mac-2/Galectin-3 (M3/38 unconjugated, BLD-125401, 1:100), Osx (rabbit polyclonal, sc-22536-R, 1:200), Runx2 (EPR14334 conjugated AF647, ab215955, 1:100), Sox9 (EPR14335 unconjugated, ab185230, 1:200), VEGFA (rabbit polyclonal unconjugated, ab46154, 1:100). Primary antibodies were stained with secondary antibodies when unconjugated (1:500, Thermo Fisher, anti-rat conjugated AF488, A21208; anti-rat conjugated AF546, A11081; anti-rat conjugated AF594, A21209; anti-rabbit conjugated AF488, A21206; anti-rabbit conjugated AF546, A10040; anti-rabbit conjugated AF647, A31573; anti-goat conjugated AF647, A21447; or streptavidin conjugated AF546, S11225). Samples were washed between steps and after staining with PBS/0.1 % Tween 20 for 3 × 5 min. Stained samples were kept in PBS for 5 min and embedded using aqueous mounting medium (Fluoromount, Thermo Fisher, MA, US) and analyzed microscopically within 6 days. Simultaneous detection of Ly6C and Ly6G was considered to indicate presence of the Gr-1 protein.

Movat's Pentachrome images were taken with a light microscope in a 2.5 × magnification and the program AxioVision (both Carl Zeiss Microscopy GmbH, Germany). For **Figure 2**, images (CD31 & Emcn) were taken with a Keyence microscope (BZ 9000) using a 10x or 4x objective. All other images were acquired at a Zeiss LSM880 in tile scan mode at a resolution of 2048 × 2048 using a 20x objective, unless specified otherwise. For display, pictures were background subtracted and contrast adjusted using ImageJ 1.52i.

### Image Analysis

Image analysis of cell and tissue distribution (**Figure 2**) was performed with ImageJ and an own developed pipeline which has been published and described in detail previously (21). Mean intensity was determined with ImageJ within the marked ROIs distinguishing between the gap, the adjacent to the gap and the bone marrow area. Mean intensities were normalized to the maximum intensity of the image.

Quantification was performed in *CellProfiler 3.1.8*. (36) (Supplementary Figure 1). Macrophage subsets were described via the co-localization of identified CX3CR1^+^, Gr-1^+^, and F4/80^+^ objects. CX3CR1^+^F4/80^+^Gr1^+^ objects, representing cells were divided by roundness based on object shape features (FormFactor, Perimeter, Min Feret Diameter, Supplementary Figure 5). Objects were only considered cells when they overlapped with a nucleus (DAPI) signal. In a second pipeline localization of F4/80^+^ macrophages towards the Emcn^+^ endothelium was analyzed by examination of the direct (≤3.5 μm) and distant (>7 μm) neighborhood of the subset. Localization of the identified subsets was analyzed by counting the identified objects in regions of interests (ROIs). ROIs for the osteotomy gap were determined as shown in **Figure 5E**. For neighborhood analysis and frequency determination (**Figures 3E,F**) either Emcn^hi^ or Emcn^lo^ areas within the osteotomy gap or distant thereof were encircled freehand using ImageJ 1.52i, respectively (**Figure 3D**). Areas close to Sox9^+^ chondrocytes were excluded as they did contain few, if any macrophages. Emcn^hi^ areas largely overlapped with F4/80^hi^ areas.

#### Object Identification and Neighborhood Analysis

For the segmentation, the model-based approach was applied (37). *Otsu* was used as a segmentation algorithm calculating the thresholds for the object edge identification. Mid-level pixels were assigned to the background. The threshold calculation was performed adaptively, which allows adaptation of the threshold to different image sub-regions. For each channel, the parameters were manually optimized by visually inspecting the segmentation results. To aid the segmentation, channel specific object ranges were estimated (e.g., nuclei = 3.1–12.5 μm). Clumped cells were separated based on their intensity distribution. Only partly visible cells touching the border of the image were removed from the segmentation process. Nuclei, CX3CR1, F4/80, and Emcn primary objects were identified based exclusively on the image. Due to the dense packing of Gr-1^+^ cells, segmentation was performed via secondary object identification with prior segmented nuclei as cellular reference. The segmentation was channel dependently refined based on various object features (Supplementary Figure 1B). Objects features were extracted on the boundaries of the objects for shape (e.g., perimeter, area, radius; Supplementary Figure 5), and intensity, computed from the signal intensity values of the object area within a defined image channel. Filter and thresholds were determined by visual evaluation of specific phenotypic measurements of individual objects within the image and the global distribution of the phenotypic measurement within the whole image.

#### Spatial Polarization Scoring

Bone orientation was determined using Movat's Pentachrome overview images. Scores were determined for individual channels (DAPI, F4/80, or CD31). Scores were determined considering the staining intensity and abundance of the signal in parts of the bone marrow not affected by the injury (homeostatic control), on the same slide in at least 400 μm distance from the injury site. Scores were: −2 for the absence of staining in the area or reduced signal abundance and intensity throughout the entire area.; −1 for signal either reduced in intensity but displaying comparable abundance, or reduced abundance and comparable intensity; 0 for appearance that resembles homeostasis (unaffected bone marrow in the same section); 1 for higher intensity at comparable or increased abundance that localized only partially along the line between the gap and the adjacent tissue. Adjacent tissue is tissue along the contour between bone marrow and hematoma, bone fragments, and callus; 2 for higher intensity at comparable or increased abundance along the entire length of the line between the gap and the adjacent tissue or in large areas extending >400 μm distance from the gap in the bone marrow tissue. Regions often showed enlarged and unstructured vessel organization, with very bright F4/80 cells between the endothelial lines. Samples were considered spatially polarized when the scored values for F4/80 and vessel marker of proximal and distal to the fracture gap resulted in a difference >2 favoring one side (proximal, distal). Samples which scored a difference of <2 were considered neutral. In total *n* = 20 samples were analyzed, 9 samples scored values other than 0 of which 2 samples were considered neutral, 1 polarized proximally and 6 polarized distally (Supplementary Figure 4A).

### Longitudinal Intravital Microendoscopy of Murine Osteotomy

As a GRIN lens system, we used custom singlet GRIN needle microendoscope (length ca. 5.07 mm, diameter = 0.60 mm; NEM-060-10-10-850-S-1.0p, GRINTECH Jena, Germany). The GRIN lens was glued into the lens tubing to a final penetration depth of 650 μm when screwed into the fixator plate. CX3CR1:GFP mice were anesthetized and mounted to the microscope as previously described (10). Qtracker 655 Vascular Label (Thermo Fisher, MA, US) were injected and images were acquired (505 × 505 px, 500 × 500 μm, unidirectional, line average 4, step size 4.5–6.5 μm, ca. 18 steps, stack time 60 sec). Image stacks were loaded into Imaris 9.3.0 software (Bitplane Zürich, Switzerland), median filter (3 × 3 × 3) was applied. Videos were exported (1024 × 1024, 4 fps) and images were angled maximum intensity projections. Individual mice were measured at 940–950 nm on day 2, 3, 4, 5 after osteotomy.

### Statistical Analysis

Statistical analysis was carried out with GraphPad Prism V.5 or V.8 software. All values are expressed as the mean ± SD if not stated otherwise. Mann-Whitney *U*-test, Wilcoxon-signed rank test and Friedman test with Dunn's *post-hoc* test were mainly used, since Gaussian distribution was not expected due to inter-individual variations. A *p* < 0.05 was considered statistically significant. Image analysis was blinded for time points.

## Results

### Emcn^hi^CD31^hi^ Endothelium Is Present During Endochondral Bone Formation in the Osteotomy Gap

**Figure 1:**
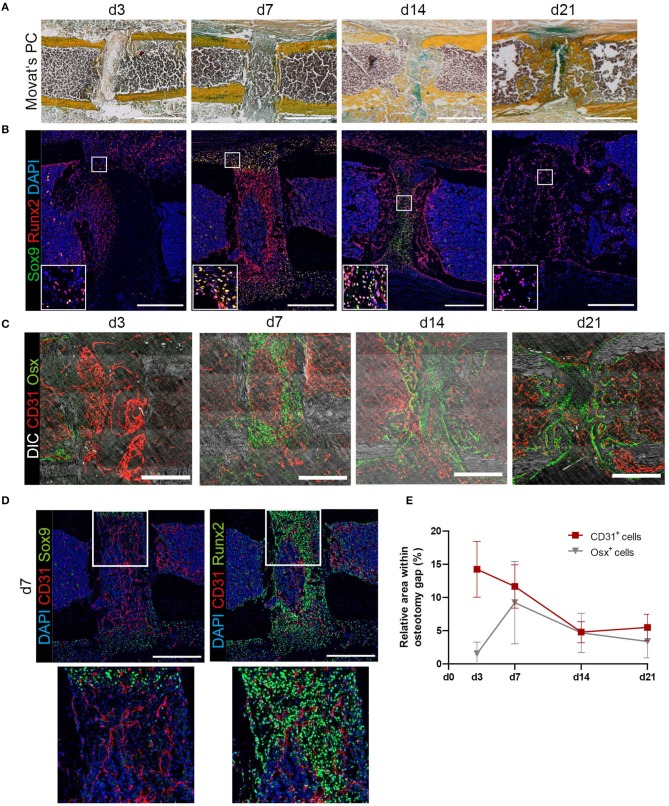
Histological characterization of tissue formation and distribution of mesenchymal progenitor cells, osteoblasts and endothelial cells during bone regeneration in a mouse-osteotomy model. **(A)** Movat's Pentachrome staining indicates the phases of bone regeneration over 21 days. Scale bars = 500 μm. **(B)** Sox9 and Runx2 immunofluorescence staining for progenitor cells undergoing differentiation at day 3, 7, 14, and 21 indicating double-positive cells at day 7 and high numbers of Runx2^+^ cells within the fracture gap at day 14 and 21. Exemplary images of *n* = 3–6 per time point. Scale bars = 200 μm. Insets show enlarged representative areas marked by white frames. **(C)** Exemplary images at day 3, 7, 14, and 21 displaying the distribution of CD31^+^ ECs and Osx^+^ osteoprogenitors/pre-osteoblasts revealing close proximity between CD31^+^ endothelium and Osx^+^ cells. Scale bars = 200 μm. **(D)** Exemplary images for Sox9 or Runx2 and CD31 staining at day 7. Insets show enlarged representative areas marked by white frames. While Runx2^+^ cells can be found closed to endothelial cells, Sox9^+^ cells can be found in not yet vascularized areas. **(E)** Quantification of cellular compartments present in the fracture gap during regeneration. Endothelial cells (ECs; CD31^+^) and osteoprogenitors/pre-osteoblasts (Osx^+^) were quantified based on the relative presence of positive pixels with the respective markers in immunofluorescence images supporting the descriptive analysis on the spatiotemporal distribution of progenitor cells, osteoblasts and endothelial cells over time. Data show mean ± SD for *n* = 3–6.

Fracture healing consists of consecutive phases. Progression into each phase depends on the undisturbed and error-free course of the respective previous phase (38). In the mouse-osteotomy model used in this study, residuals of cells in the fracture hematoma are visible in Movat's Pentachrome staining at day 3, while the osteotomy gap is filled with a mixture of bone marrow cells including hematopoietic cells at day 7, and endochondral bone formation occurs between day 14 and day 21 (Figure 1A). Using immunofluorescence histology, a positive signal for CD31 (Platelet endothelial cell adhesion molecule 1) is seen in elongated cells inside the fracture hematoma, which include no detectable nuclei (Supplementary Figures 2A–C), probably indicating that these residuals represent endothelial cells that may have lost their integrity. In order to further characterize the cellular composition within the fracture area, immunofluorescence staining for key transcription factors of mesenchymal differentiation was performed on serial sections of the same bones (Figure 1B). Already at day 3, osteoblast progenitors characterized by nuclear expression of Runt-related transcription factor 2 (Runx2) are dispersed in the fracture gap. By day 7, their number has increased, leading to a dense population of the gap, whilst sparing a region in the center. The peripheral borders of the gap and adjacent periosteal regions are populated by cells co-expressing Runx2 and SRY-box transcription factor 9 (Sox9) in the nucleus, indicating their potential for either osteogenic or chondrogenic differentiation (Figure 1B). This is in accordance with the blue-greenish color in corresponding regions of Movat's Pentachrome staining, indicative of cartilaginous tissue (Alcian blue positive). At day 14, Runx2^+^ cells are found to localize on both sides of the gap. Single Runx2^+^ cells are surrounded by areas characterized by the presence of only a few nuclei, in line with the presence of mineralized bone in those areas, as visualized by Movat's Pentachrome staining (compare: Figures 1A,B). Sox9^+^ cells are exclusively present in the center of the gap at this time point. Notably, the majority of these cells do not co-express Runx2. In contrast, at day 21, a time point when mineralization of the gap is complete, the progenitors identified in this area are almost exclusively Runx2^+^. In a next step, Osterix (Osx) and CD31 stainings were performed on serial slides from the same individuals. Direct comparison reveals that at day 3, only some Runx2^+^ cells are also Osx^+^, indicating various degrees of osteogenic differentiation. From day 7 on, Runx2^+^ cells are also positive for Osx^+^, consistent with ongoing maturation of osteoblasts. Osx^+^ osteoblasts localize in the fracture gap alongside existing and newly formed bone, as indicated by differential interference contrast (DIC) signal. Sox9^+^ cells can be found at day 14 and 21 in areas with cartilage and close to Osx^+^ cells, indicating ongoing further differentiation and mineralization, which is accompanied by vascularization. For further quantitative analysis of the vasculature sprouting into the gap, we defined the gap as a rectangular region between the cortical ends and analyzed CD31^+^ vessels and Osx^+^ bone cells in this region (Figure 1C). At day 3, CD31^+^ matrix (residuals of the fracture hematoma) and some CD31^+^ cells are present (compare to Supplementary Figures 2A–C), while Osx^+^ cells enter the osteotomy gap at later time points, between day 7–21 (Figures 1C,E). At later phases of fracture healing, endothelial cells displaying the phenotype of type H vessels (Emcn^hi^CD31^hi^) closely associate with Osx^+^ osteoprogenitors/pre-osteoblasts (Figure 1C). Investigating the co-localization of Runx2^+^ and Sox9^+^ cells with respect to CD31^+^ endothelium reveals a close localization of Runx2^+^ cells around vascularized areas, while Sox9^+^ cells are only found in less vascularized areas (Figure 1D). Our results confirm a close relationship between vessel formation and osteoprogenitors/osteoblasts and extend previous reports of this phenomenon, which focused on bone development (7), to a regenerative scenario.

### Localization and Morphologic Characterization of Emcn^hi^CD31^hi^ Type H Endothelium

**Figure 2:**
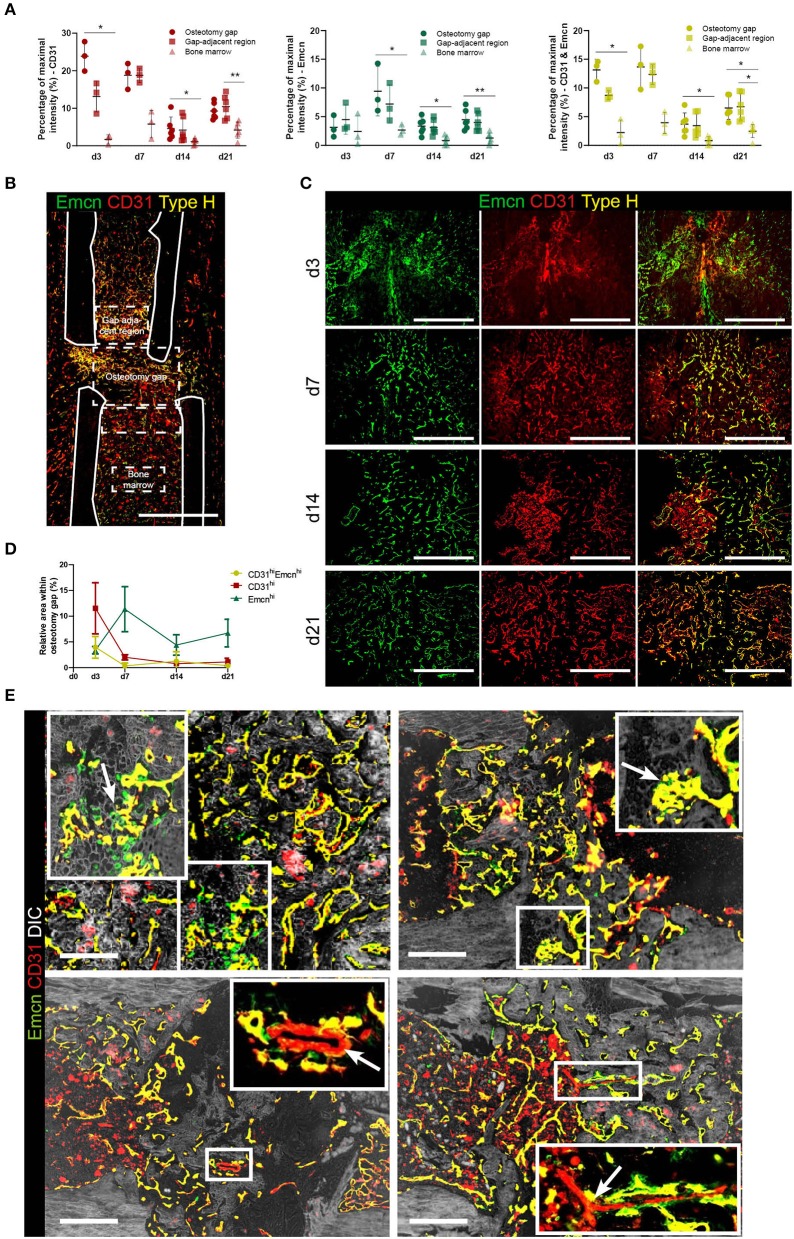
Vessels formed during fracture healing show characteristics of type H endothelium and are embedded in mineralized tissue. **(A)** Intensity analysis of Emcn and/or CD31 in the fracture gap. Mean intensities were normalized to the maximum intensity present in each image. Mean intensities were normalized to 780 the maximum intensity present in each image. Data are shown as scatter dot plot with mean ± SD while one dot is representative for one slide of one individual animal. Friedman test with Dunn's *post-hoc* test were performed to determine statistical differences; *p*-values are indicated with *p < 0.05, **p < 0.01. **(B)** Emcn^hi^ CD31^hi^ endothelium shows spatial differences in abundance throughout the bone. Exemplary image showing the definition for the osteotomy gap, the gap adjacent regions and the bone marrow area, as used in our analyses. Scale bar = 200 μm. **(C)** Images of Emcn^hi^ CD31^hi^ endothelium at day 3, 7, 14, and 21. Data are representative for *n* = 3 (d3/7) and *n* = 6 (d14/21). **(D)** Quantification of areas occupied by Emcn^hi^ CD31^hi^ type H endothelium during the time course of bone healing. Data are shown as Mean ± SD. **(E)** Emcn and CD31 staining combined with phase contrast images (DIC) in the soft to hard callus transition of fracture healing highlight morphological characteristics also present in growth plate bone growth. Upper panels: invasion of vessels in cartilaginous tissue and type H vessel-like budding is indicated by arrows. Lower panels: CD31^+^ only vessels are assumed to be arterioles (arrows) (7). Scale bars = 200 μm.

While Emcn^hi^CD31^hi^ endothelium can be found directly in the osteotomy gap and in the adjacent tissue, Emcn^lo^CD31^lo^ endothelium is prominent in bone marrow regions not affected by the injury. Pixel intensity analysis confirms the differences between the Emcn^+^ and/or CD31^+^ cells in and adjacent to the osteotomy gap, compared to the bone marrow (Figures 2A,B).

We further characterized the type H vessels in the fracture gap (Figures 2C,D). During vascularization of cartilaginous tissue, invading vessel buds (distal loops), previously described as a morphological criterion for type H endothelium (7), can be identified (Figure 2E, first row marked by arrows). Furthermore, CD31^+^ arterioles, which are negative for Emcn, are found within the osteotomy gap. However, a second morphological criterion described to be typical for type H vessels in the growth plate areas, namely their columnar structure, does not appear during regeneration, suggesting that this ordered arrangement is a specific feature of bones undergoing longitudinal growth. Taken together, our data reveal the appearance of Emcn^hi^CD31^hi^ in the fracture gap, which share additional distinct morphological features with the previously described type H vessels, although they are not organized in columnar structures. This finding suggests an important role of this osteogenic vessel type not only during bone development, but also during regenerative processes of the bone.

### M2-Like Macrophages Localize Preferentially Extramedullary

**Figure 3:**
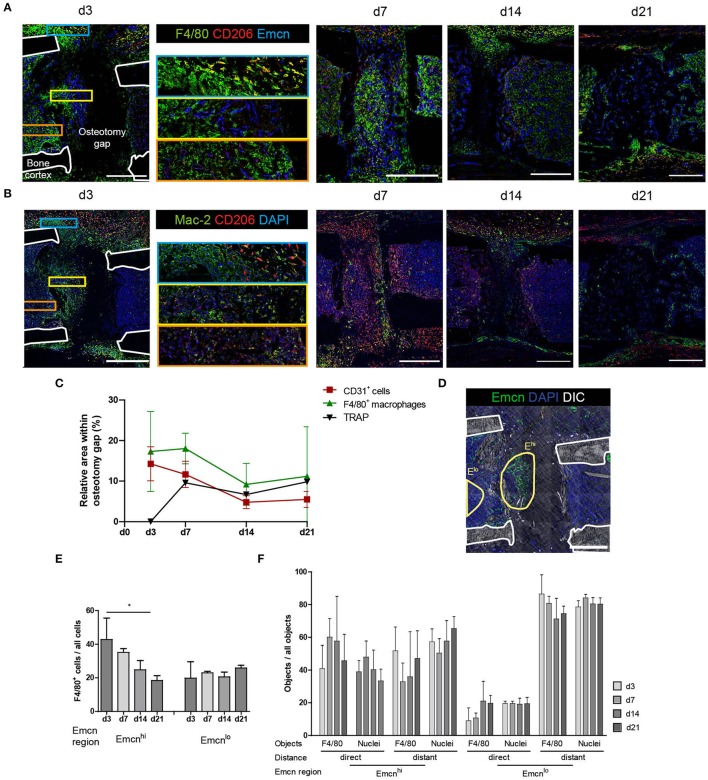
Distinct localization of macrophage subsets during bone regeneration indicates location-dependence of their functions. **(A)** Immunofluorescence staining of F4/80, CD206, and Emcn shows the abundance of F4/80^+^ cells in the osteotomy gap. They localize close to Emcn^hi^ endothelium. F4/80^+^CD206^+^ M2-like macrophages primarily localize in extramedullar areas (blue inset). F4/80^hi^ cells localize in proximity to Emcn^hi^ endothelium (yellow inset). Endothelium shows CD206-positivity in proximity to the gap (orange inset). Scale bars = 500 μm. **(B)** Immunofluorescence staining reveals spatiotemporal distribution of CD206^+^ and Mac-2^+^ cells. CD206^+^ M2-like macrophages were Mac-2^+^ at d3 and single-positive by d21. Scale bars = 500 μm. **(C)** Pixel-based area analysis of immunofluorescence images of CD31, F4/80, and TRAP show a reduction of CD31 and F4/80 signals over time and an increase in osteoclasts. **(D)** Object-based analysis for **(E,F)**, based on regions within the osteotomy gap which show high or low expression of Emcn (Emcn^hi^ vs. Emcn^lo^). Scale bar = 500 μm. **(E)** F4/80^+^ cell frequency in the Emcn^hi^ region decreases between d3 and d21 to levels of the Emcn^lo^ region. Mann Whitney test, two-tailed, *p = 0.0476. **(F)** Proximity analysis performed by object-based quantification in two distances from the endothelium. Data was normalized to the overall number of the object population in the respective region. Compared to the Emcn^lo^ region, more objects (F4/80^+^ or nuclei) localize in proximity to the endothelium in the Emcn^hi^ region. Cells were considered in proximity to each other when their distance amounted to that equivalent to less than half of a nucleus diameter (<3.5 μm), in order to include cells which are either in contact with or in the direct vicinity of vessels. Cells were considered distant were located further than one cell layer (>7 μm) apart from each other. Data are representative for *n* = 3 (d3/7) and *n* = 6 (d14/21).

In order to determine the role of macrophages in the context of vascularization during bone regeneration, we analyzed macrophage subsets and their spatial localization relative to type H vessels. Based on the pan-macrophage marker F4/80, which includes also osteomacs, we defined M1- and M2-like macrophages labeled by CD80 and CD206 (Mannose receptor), respectively, as well as Mac-2 (Galectin-3), a marker associated with pro-inflammatory macrophages (22). Over the course of endochondral bone regeneration, F4/80^+^ cells localize throughout the bone marrow, as well as on the border to bone surfaces, accumulating in various areas (Figure 3A). At day 3 post-osteotomy, high numbers of F4/80^hi^ cells localize in periosteal regions adjacent to the osteotomy gap. They can be found periosteal in varying numbers throughout the regeneration process, until the bone remodeling phase at day 21. At day 14, F4/80^hi^ cells are found exclusively in periosteal regions, whereas they are found in both the medulla and the periost at day 21. Extra-medullar F4/80^+^ macrophages are positive for the anti-inflammatory M2(a) macrophage marker CD206 (Figure 3A, blue framed inset). Those M2-like cells are not abundant in the marrow or at sites of vascularization, where expression of CD206 is generally lower and macrophage morphology rather ramified (Figure 3A, yellow and orange framed insets). CD206 expression is also found in cells resembling the morphology of endothelia (Emcn^+^), which are F4/80^−^ throughout the regeneration process as well as under homeostatic conditions (Figure 3A, yellow framed inset; Supplementary Figures 3C,D). M1-like macrophages, defined by expression of CD80, are extremely rare and only few are found at day 3, with their abundance comparable to control tissue (Supplementary Figures 3B,D). Using the marker Mac-2 in addition, almost all CD206^+^ macrophages are found to be positive for Mac-2 at day 3, however, not all Mac-2^+^ cells are CD206^+^ (Figure 3B). Mac-2 positive cells localize in the fracture gap and in proximity to the bone surfaces. Over the course of regeneration, double-positive Mac-2^+^ and CD206^+^ cells vanish and cells which are single positive for each of the two markers are detected at day 21, a time point when remodeling is ongoing (Figure 3B). Quantitative pixel-based area analysis of F4/80 macrophages, CD31 and TRAP shows that CD31^+^ and F4/80^+^ cells are reduced over time, while the number of TRAP^+^ cells (osteoclasts or activated macrophages) increases (Figure 3C; Supplementary Figure 3A).

CD206^+^ macrophages are present throughout the healing process with no apparent preferential localization towards the vasculature, and they are mainly Mac-2^+^ in the early phase of regeneration. Over the course of regeneration, F4/80-expression decreases in regions adjacent to the callus, and at later time points only few areas, which contain cells expressing F4/80 at intermediate levels, are observed.

### F4/80^+^ and CD80^+^ Cells Produce VEGFA During Early Regeneration and Osteoblasts and Chondrocytes Are the Main Producers at Later Time Points

**Figure 4:**
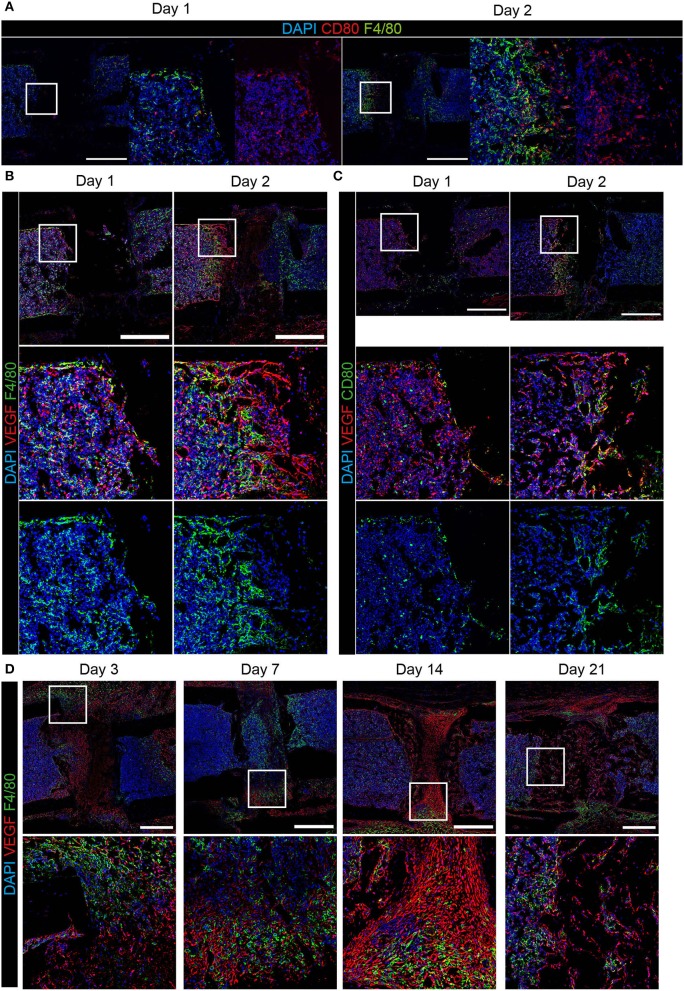
Expression of VEGFA in CD80^+^ cells and bone forming areas at the osteotomy site. Immunofluorescence staining over the course of regeneration shows **(A)** pro-inflammatory CD80^+^ cells adjacent to the osteotomy gap, which are in part F4/80^+^ in the early phase (at day 1 and 2). **(B)** F4/80^+^ cells were predominantly VEGFA^−^. **(C)** CD80^+^VEGFA^+^ cells localize at the interface between the hematoma and surrounding tissue. **(D)** In areas of bone formation VEGFA expression is high, but F4/80^+^ cells re VEGFA^−^ during progressing bone regeneration until day 21. Scale bars = 500 μm. *n*(day 1) = 3, *n*(day 2) = 1.

Since we could not find substantial amounts of CD80^+^ cells at day 3 post-osteotomy, we analyzed the regeneration at day 1 and day 2. Some CD80^+^ cells were found at day 1, and they were abundant in higher numbers at day 2 post-osteotomy (Figure 4A). Some of those are F4/80^+^CD80^+^ macrophages, which localize in areas adjacent to the osteotomy gap. CD80^+^ cells are located directly at the injury site (Figure 4A, day 2). Macrophages are known to support vascularization via VEGFA during tissue regeneration, which is why next, we analyzed VEGFA using immunofluorescence histology over the time course of bone regeneration (Figures 4B–D). VEGFA is not expressed by F4/80^+^CD80^−^ macrophages throughout the entire process, but CD80^+^F4/80^−^ and a fraction of CD80^+^F4/80^+^ cells express VEGFA. This expression was restricted to very early time points, namely day 1 and day 2 (Figures 4B,C). From day 3 on, VEGFA is found to be expressed in the fracture gap and along bone surfaces (endosteal, periosteal) and in the bone forming areas (Figure 4D). The signal for VEGFA at day 14 is pronounced in areas, which also contain Sox9^+^ and Runx2^+^ cells (Figures 1B, 4D). Taken together, VEGFA expression is observed in CD80^+^ cells, including a fraction of F4/80^+^ cells, in the early phase until day 3, after that, VEGFA is expressed predominantly by osteoblasts and chondrocytes in bone forming areas.

### Cells Within the Osteotomy Gap Localize in Proximity to the Endothelium

High abundance and expression of the vascular markers CD31 and Emcn is observed in both, the damaged tissue in the early regeneration, and in bone forming tissue over the subsequent course of regeneration in the osteotomy gap (Figure 2C). These areas rich in type H vessels, simultaneously contain F4/80^hi^ cells (Figures 3A,D). In order to evaluate the proximity in localization between macrophages and type H endothelium, we defined two regions for each sample, the Emcn^hi^ region and the Emcn^lo^ region (Figure 3E). Objects in these regions were segmented based on marker expression of Emcn, F4/80 and DAPI. Over the course of regeneration, similar frequencies of 25.0 ± 11.6 % F4/80^+^ cells are present in both, the Emcn^hi^ and Emcn^lo^ regions. The frequencies of F4/80^+^ cells decrease significantly from day 3 until day 21 post-injury (Figure 3E), confirming the qualitative results from Figure 3C. Next, we analyzed the position of macrophages relative to the endothelium, and found that, a two- to three-fold higher number of objects (F4/80^+^ objects or identified nuclei, which serve as a control for all cells) localize in proximity to the endothelium in the Emcn^hi^ region as compared to the Emcn^lo^ region (Figure 3F). Cells are defined as “in proximity” when they are located less than half a nuclear diameter in distance (<3.5 um) away from the endothelium.

**Figure 5:**
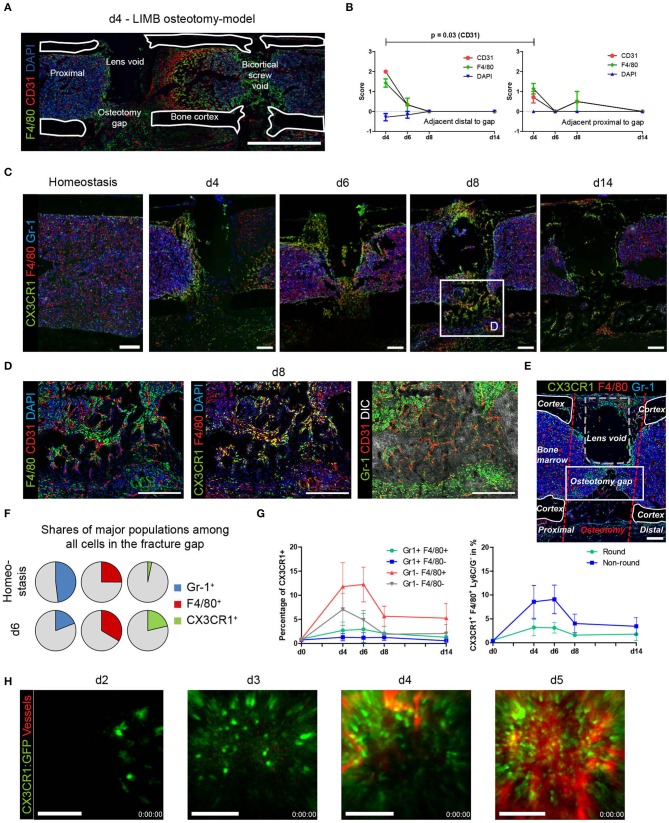
Quantitative analysis and LIMB imaging demonstrate high abundance of the myeloid CX3CR1^+^ F4/80^+^ cell subset preceding vascularization. **(A)** Overview image of a section from a whole bone taken at day 4 after LIMB osteotomy. The LIMB osteotomy-model model uses a gradient refractive index lens which is removed before bone sectioning thus a *lens void* remains. Vessels and macrophages polarize distally in this example. **(B)** Scoring of CD31 and F4/80 allows quantification of polarization in proximal or distal orientation with respect to the fracture gap. Scoring of DAPI staining was used as internal control. Wilcoxon matched-pairs signed rank test. Data are representative for *n* = 7 (d4), *n* = 6 (d6), *n* = 4 (d8), and *n* = 3 (d14). **(C)** Immunofluorescence images of sections from bones showing the presence of myeloid subsets at various time points after LIMB osteotomy. CX3CR1:GFP^+^ and F4/80^+^ cells negative for Ly6C/Ly6G (Gr-1), accumulate at and invade into the fracture gap. Scale bars = 200 μm. **(D)** Inset of the area of d8. The majority of myeloid cells are CX3CR1^+^F4/80^+^ and localize in distance from Gr-1^+^ cells. Scale bars = 200 μm. **(E)** Quantitative analysis is performed in the rectangular region of the fracture gap between corticalis, osteotomy cuts, and lens void (osteotomy gap). Scale bar = 100 μm. **(F)** Quantitative, object-based analysis of the osteotomy gap for Gr-1, F4/80, CX3CR1:GFP show a decrease of Gr-1^+^, an increase of F4/80^+^ and massive increase of CX3CR1^+^ cells. Results of 20 pooled samples. **(G)** Object-analysis among the CX3CR1^+^ cells reveals that Gr-1^−^F4/80^+^ account for the majority of cells present in the gap over the whole time course of regeneration (left panel). Of those, the majority were non-round cells (right panel). **(H)** Intravital two-photon LIMB-microscopy of the osteotomy gap in an individual CX3CR1:GFP mouse. CX3CR1:GFP^+^ cells invade the osteotomy gap in the displayed field of view (250 × 250 μm) at day 2–3 and fully populate the region by day 3–4. Simultaneously, numerous CX3CR1^+^ cells precede the vascularization in space and time. Vasculature was made visible using intravenous injection of Qtracker 655. Scale bars = 100 μm. Data are representative of 5 mice analyzed.

During analysis of immunofluorescence staining in samples of osteotomized, LIMB-implanted bones, we noticed a transient, strongly polarized distribution of newly formed vessels to the distal area of the osteotomy gap (Figure 5A; Supplementary Figure 4A). A similar polarization occurs in samples of the osteotomy-model (Supplementary Figure 4B). In some cases, this phenomenon is accompanied by a variation in cell density in the respective area, as shown by differences in the abundance of the DAPI signal. Scoring according to the criteria described in the methods section for the abundance and intensity of the markers in the gap-adjacent regions reveals that CD31^+^ vessels are significantly brighter and more abundant adjacent distally to the gap, as compared to the proximal site (Figure 5B). This spatial polarization with respect to bright CD31^+^ vessels is pronounced at day 4 and decreases quickly afterwards (Figure 5B). A similar trend is observed for F4/80^+^ cells in the same regions (Figure 5B). Taken together, these analyses reveal a transient, directional spatial polarization (regarding the localization from the fracture gap) of CD31^hi^ vessels, adjacent to the fracture gap.

### CX3CR1^+^ F4/80^+^ Macrophages Precede Vascularization

In order to understand the dynamics of macrophages during bone regeneration, we took advantage of the fractalkine receptor (CX3CR1) reporter mouse strain CX3CR1:GFP. These mice were implanted with a Gradient Refractive INdex (GRIN) lens, enabling longitudinal imaging of the bone marrow during regeneration (10). At the same time, these mice underwent osteotomy surgery. Since the first wave of vascularization occurs during resolution of the fracture hematoma, bones that had been treated using the procedure described were fixed and analyzed at day 4, 6, and 8 using immunofluorescence histology. In order to be able to exclude monocytes and granulocytes from the macrophage analysis, we stained sections using antibodies against Ly-6C/Ly-6G (Gr-1). We find that almost all F4/80^+^ macrophages in the osteotomy gap are also CX3CR1^+^ (Figures 5C,D, yellow), confirming that the reporter mice are suitable for tracking macrophage dynamics during bone regeneration. GFP^+^ cells are present within the osteotomy gap at early time points and become less abundant over time. They locate in close proximity to CD31^hi^ endothelium, do not express Gr-1 (Figure 5D), excluding the possibility that some of them are granulocytes, which are also described to be CX3CR1^+^. We analyzed the osteotomy gap between the cortices and the bone marrow as described in Figure 5E. At day 6, of all identified cells in the osteotomy gap, the CX3CR1^+^ cells display the highest increase in cell number, as compared to homeostasis. A minor increase is detected in F4/80^+^ cells and a strong reduction of Gr-1^+^ cells is observed compared to homeostasis (Figure 5F). The space is largely occupied by F4/80^+^ myeloid cells, which are not monocytes (Gr-1^−^). The strongest increase in cell number is observed in the CX3CR1^+^Gr-1-F4/80^+^ subset, which make up to 12% in ratio to all nuclei at day 6 (Figure 5G). Almost 6% of CX3CR1^+^ objects are F4/80^−^ and only few are Gr-1^+^. Morphologically, the cell phenotype is of non-round shape (Figure 5E, Supplementary Figure 5). We then longitudinally sampled time-lapse videos with two-photon microscopy from individual mice over the course of the early regeneration process. On the first and second day, almost no signal is detected inside the osteotomy gap (data not shown). On day 3, individual CX3CR1^+^ cells enter the hematoma (Figure 5Hd3). Those cells display a non-round, but non-ramified shape and move through the tissue (Supplementary Movie 1). A front of CX3CR1^+^ cells forms at the edge of the field of view at day 4, containing both round and non-round cellular phenotypes, forming a dense, partially resident population which expands into the entire field of view (Figure 5H, Supplementary Movies 2, 3). This invasion is accompanied by the occurrence of perfused vessels that expand, following the CX3CR1^+^ cell front until the field of view appears vascularized (Supplementary Movie 4). Under full vascularization, sessile CX3CR1^+^ cells localize towards the vasculature and motile cells can be observed to move along the endothelium (Supplementary Movie 3). Generally, the abundance of CX3CR1^+^ in the gap increases with progression of the regeneration process (Figure 5F, Supplementary Movies 3, 4).

These results indicate that most cells in the fracture gap are myeloid, non-monocytic, non-granulocytic, non-round CX3CR1^+^ which invade the osteotomy hematoma starting day 2–3, become gradually sessile in the fracture gap at day 3–4, where they precede the vasculature until it becomes fully perfused. After vascularization, CX3CR1^+^F4/80^+^ cells persist until the onset of the remodeling phase.

## Discussion

Vascularization is pivotal to the success of complete, scar-free bone regeneration. Here, we show that after bone injury, type H endothelium evolves in the bone formation region and persists throughout the entire bone regeneration process. Our findings show that the phenotype of type H endothelial cell structures described in endochondral long bone growth, is also reflected in bone regeneration. Emcn^hi^CD31^hi^ endothelium shows association with Osx^+^ osteoblasts and displays typical features that can be observed in the growth plate during longitudinal growth, such as invading vessel buds and arch-like structures described before (7). Other than their counterparts in the growth plate, type H vessels generated during regeneration are not columnar. However, this feature is probably not inherent to type H vessels, but merely the result of the highly ordered structure of chondrocytes in the metaphyseal areas. These chondrocytes are organized into columns that are produced in the growth plate, and probably impose their structure on the type H vessels in these areas. In contrast, the tissue structure surrounding type H vessels in a fracture gap appears more disorganized. We have previously shown that the amount of type H vessels can be used as a measure of fracture healing progression (21). Ramasamy et al. reported the effect of shear stress in the vascular formation during bone development, therefore it would be interesting to analyze whether interfragmentary, compressional mechanical cues also act on the formation and structure of type H vessels in fracture healing (39).

We found an extensive staining of CD31 in the gap, which dominates the initial hematoma phase (until day 3–4). This staining seems not to be associated with intact cells, since no nuclei are present. It is possible that CD31^+^ cells are present in the early fracture hematoma during the first hours after injury and undergo cell death due the hypoxic microenvironment, including a low pH and high lactate level in the tissue, and that the staining pattern observed represents dead endothelial cells.

Based on our results, and similar to a previously reported model in sheep (6), we propose that two phases of vascularization occur during bone regeneration also in mice. The first wave of vascularization occurs during disintegration of the fracture hematoma, where vascular sprouts from existing endothelial cells enter into a hypoxic environment. It occurs between the initial injury and day 6, until the entire volume is fully vascularized for soft callus formation. We observe this phase histologically and by intravital microscopy. After the first wave, and once the callus becomes calcified, we assume that the vascular network within hard callus and bone is remodeled in a second phase, based on the changes that happen during tissue reorganization. It has been described that most of the progenitor cells that contribute to fracture repair immigrate from the periosteum (40). In addition, these progenitor cells either express Sox9 or Runx2, which are regulated by direct repression of the opposing pathway (via β-catenin expression) and define further differentiation into chondrocytes or osteoblasts, respectively (41). However, several studies show the importance of Sox9 expressing progenitor cells that support mineralization and osteogenesis within the fracture gap (42–44). At day 7, we observe a co-localization of Sox9 and Runx2 (Figure 1B), it can be speculated that co-expression of both transcription factors marks a switch in the genetic program, from uncommitted pre-osteoblasts to chondrocyte differentiation, similar to what has been shown in bone development (45). Alternatively, Sox9 expressing cells might differentiate into osteoblasts, as described previously (42–44). Our results show close proximity between osteoprogenitors (Runx2^+^) or osteoblasts (Osx^+^) and CD31^+^ endothelium (Figures 1C,D). Endothelial cells, which display the phenotype of type H vessels (Emcn^hi^CD31^hi^) closely associate with Osx^+^ cells in this osteotomy (= cortical defect) model. Type H endothelia have been previously described to be present in the metaphyseal areas of young animals, where they promote bone growth (7). The results presented here indicate a similar crosstalk to occur in endochondral osteogenesis during regeneration.

The large extent of angiogenesis, which occurs in the fracture gap, raises the question for factors and cell types which trigger this event. In the growth plate, hypertrophic chondrocytes produce high amounts of VEGFA, due to the hypoxic microenvironment in those areas, in which HIF stimulates VEGFA expression (7). In a recent publication, Buettmann et al. deleted VEGFA from early osteolineage (Osx^+^) cells, mature osteoblasts and osteocytes (Dmp1^+^) as well as ubiquitously in models of cortical fractures (full and stress fractures) and a cortical defect model (drilling). Bone regeneration and periosteal angiogenesis after a cortical defect was impaired only when VEGFA was deleted either ubiquitously or from Osx^+^ cells, indicating a predominant role of osteolineage cells. After drill hole injury, however, the deletion did not lead to delayed healing, indicating that another cell type than Osx^+^ is responsible for VEGFA production and the progression of vascularization.

Macrophages have been identified to play a role in bone regeneration, as their deletion either via clodronate (24, 25) or genetically delays healing. Data available in the literature indicate multiple functions for macrophages during bone regeneration. Recently, the supportive function of F4/80^+^ macrophages during bone regeneration via enhanced osteogenesis when transplanted to aged individuals was demonstrated (46). In addition, it is known that F4/80^+^ osteomacs support osteoblast function (47). Osteoblast differentiation and mineralization have also been shown to be modulated by macrophages (48, 49). However, the role of macrophages in the vascularization of the fracture is largely unknown. Here, for the first time, we quantitatively and qualitatively map out the vascular network, relate it to the presence of macrophages at relevant time points during bone regeneration in mice and analyze presence and proximity with different approaches. We find that F4/80^+^ macrophages and type H endothelium localize at the front of damaged tissue in the first phase until soft callus formation, when their presence is reduced. In detail, our proximity analysis reveals that all cells, including F4/80^+^ macrophages in the regenerating tissue, localize much closer to the endothelium than in unaffected areas in both, the first (d3–d7 after injury) and second (d14–d21 after injury) phase of revascularization. We find CX3CR1^+^F4/80^+^ macrophages to be a predominant subset in this process. Phenotypically, these cells are for the most part non-round and ramified. They localize predominantly in a one-cell layer around the endothelium. Future work is needed to study the fate of this subset. It is possible that those cells differentiate or merge with osteoclasts and therefore promote remodeling of the bone.

Using longitudinal intravital microendoscopy of the osteotomized region, we find that CX3CR1^+^ macrophages precede the occurrence of perfused vessels into the hematoma in the first phase and remain associated to the endothelium. We show the type H endothelium to be closely associated with CX3CR1^+^F4/80^+^ macrophages, suggesting that this myeloid population is responsible for vascularization and progression of bone regeneration.

In the fracture hematoma, no chondrocytes are present, so macrophages may be a source of VEGFA in that situation. Here, we identify two major cell populations, which produce VEGFA over the course of regeneration. In the earliest phase, at day 1 and 2 post injury, CD80^+^ cells are found to be positive for VEFGA by immunofluorescence. From day 3 on, cells inside the area of bone formation and on bone surfaces show a strong VEGFA signal. Based on their localization, the proximity to bone surfaces, we assume that these cells are precursors of, and committed osteoblasts, as well as chondrocytes in cartilage (16; compare Figures 1A–C). Until day 3, among the CD80^+^ cells, we find F4/80^+^, which we consider M1-like cells, as well as F4/80^−^, which could either be a subpopulation of mature F4/80^−^ macrophages or precursors of macrophages, such as F4/80^low^ monocytes (50). In addition, other antigen-presenting cells such as B cells and dendritic cells can be CD80^+^ (51, 52). It has been previously described that VEGFA-expression of macrophages depends on the stimulation state (53, 54). Additionally, tissue resident F4/80^+^ macrophages are not known to express VEGFA constantly, but rather they interact with VEGFA-producing cells on promoting vascularization and supporting sprout fusion (29, 55). Our results support previous literature by showing VEGFA-expression from immune cells (56). Here, CD80^+^ cells were expressing VEGFA in the early phase until day 3, while the majority was F4/80^−^. CD80^+^ M1-like macrophages have not been reported to express VEGFA. It can be speculated from our data that CD80^+^ cells contribute to the presence of VEGFA within the fracture gap until bone cells differentiate and become the dominant producers of VEGFA during the bone formation and remodeling phase.

The presence of M1-like macrophages until day 3, the subsequent decrease, and the absence at later time points in our study indicates that the transition from the pro-inflammatory phase to the anti-inflammatory phase in the myeloid compartment has already taken place at day 3 in the osteotomy models analyzed here. It has been described that a switch from M1-like macrophages to an M2-like phenotype is essential for successful healing. Under chronic inflammatory conditions it is impaired and accompanied by the prolonged presence of M1-like macrophages (57). Consequently, recent research focuses on this switch to improve healing (58, 59). Interestingly, M1- and M2-like macrophages do not localize inside the fracture gap, and rather CX3CR1^+^F4/80^+^ cells participate in the initial and crucial vascularization process. Our spatial analyses reveal that CD206^+^ macrophages localize in extramedullary areas and, moreover, that they remain positive for the pro-inflammatory maker Mac-2 until d14. After that, cells that are positive for either CD206 or Mac-2 remain present until the remodeling phase. The extramedullary localization of CD206^+^ macrophages may indicate that M2-like macrophages serve functions beyond bone regeneration, for example skeletal muscle regeneration (60).

Of note, during the regeneration process, the immunofluorescence signal for Emcn^+^ in the endothelium partially co-localizes with the signal for the mannose receptor CD206. CD206 expression in endothelial cells has been previously reported to be linked to the phagocytic activity of this cell type (61). In addition, Awert et al. describe CD206 as a marker for perivascular macrophages and show that they locate closely to Emcn^+^ cells in tumors (62). CD206^+^ endothelial cells can be found in different tissues such as the liver (63, 64) or placenta (61). Future experiments need to be performed to investigate the cellular origin of the CD206 signal we observe.

In tissue areas adjacent of the fracture gap, an inhomogeneous distribution of macrophages, as well as endothelia toward the distal end of the femur (spatial polarization), is evident. We suspect that the osteotomy, which interrupts all vessels in the tissue, in combination with placement of the neighboring, distal screw (one of four screws) creates damage to the blood supply occurring from both sides of the fracture (Supplementary Figure 6). Large veins and the main sinus exit the bone at few points. However, trans-cortical vessels are predominantly responsible for the arterial blood supply in long bones, as described recently (65). Since the cortical integrity is disrupted by the osteotomy and the screw placement, the blood supply is disturbed to a high degree in a complete osteotomy model. We assume that this results in extensive tissue damage, which in turn initiates the recruitment of macrophages followed by vascularization, leading to the observed phenomenon of transient distal polarization.

Taken together, we demonstrate here that type H endothelium is present throughout the regeneration in standardized osteotomy models in mice. Osx^+^ osteoblasts as well as macrophages are present in close proximity to the vasculature, indicating an important crosstalk. M2-like macrophages are mainly found in extramedullar regions, with no obvious interconnection to the vasculature. In addition, we describe CX3CR1^+^F4/80^+^ cells to be the predominant macrophage population, which progressively infiltrates the hematoma. These macrophages precede perfused vascularization in the first phase of vascularization and reside there until remodeling takes place. A strong polarization of type H endothelium as well as of macrophages distally to the fracture gap is found in the fracture models. Our findings underline the importance of the innate immune system within the bone regeneration process by linking myeloid cells present in the fracture gap to local angiogenesis.

## Data Availability Statement

The authors declare that all data supporting the findings of this study are available within the paper and its supplementary information file. Further information is made available by the authors upon request.

## Ethics Statement

The animal study was reviewed and approved by the local animal protection authority (LaGeSo; permit numbers: G0039/16, G0111/13, and G0302/17), and were performed in accordance with the German Animal Welfare Act.

## Author Contributions

JS, AL, TG, RN, and AH: study design. JS, AL, AR, LA, MK, KS-B, and AF: data collection and analysis. JS, AL, and AH: data interpretation and writing of the manuscript. KS-B, FB, RN, TG, and GD: revising manuscript.

### Conflict of Interest

The authors declare that the research was conducted in the absence of any commercial or financial relationships that could be construed as a potential conflict of interest.
