# Supplementary material for: Spatial Distribution of Macrophages During Callus Formation and Maturation Reveals Close Crosstalk Between Macrophages and Newly Forming Vessels
Source: Front Immunol. 2019 Nov 26;10:2588. doi: 10.3389/fimmu.2019.02588 (PMC6953593; doi:10.3389/fimmu.2019.02588)
Supplement: Supplementary file 1 [file Data_Sheet_1.pdf]

Supplementary Material

Spatial distribution of macrophages during callus formation and maturation reveals close crosstalk between macrophages and newly forming vessels

Supplementary Figure 1

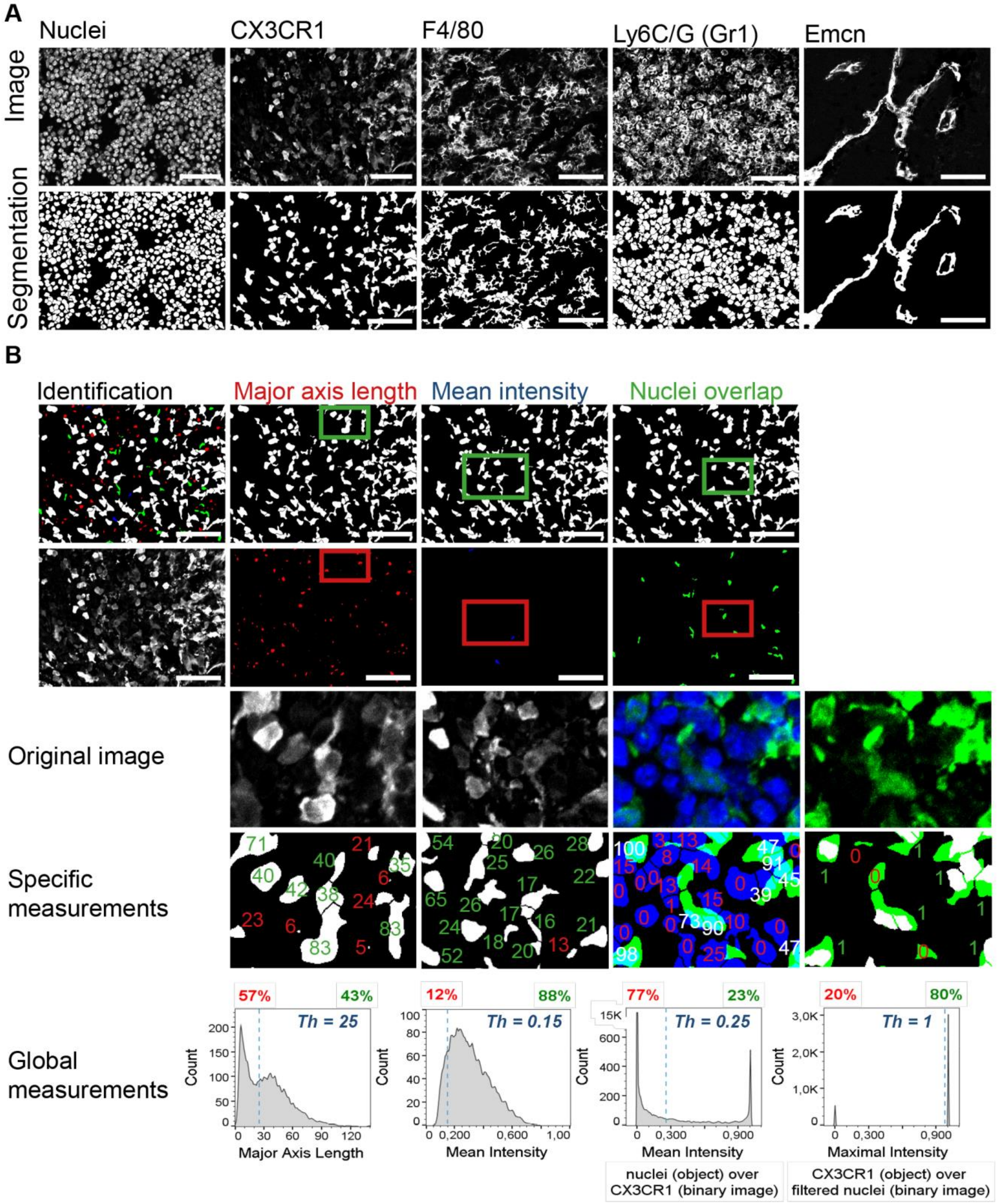

Supplementary Figure 1: Image analysis strategy for object identification. (A) Immunoflorescence images of sections of bone regeneration using the LIMB microendoscope internal fixator with their refined segmentation within CellProfiler. Scale bars = 50 µm. (B) Refinement of the segmentation of CX3CR1:GFP$^+$ cells based on various object features. Identified objects were removed based on their size (major axis length; red) and their signal intensity (mean intensity; blue). Only cells in the focus plane (i.e. with nuclei) were analyzed (nuclei overlap; green).

The thresholding (Th, blue line) was based on one hand on the visual observation of specific measurements of individual objects in relation their identification within the original image (depicted as absolute values for the major axis length and as percentages for the intensity measurements with the measurements associated with removed values in red and the other values in green). Additionally, the global measurement distribution of all objects within the image (depicted in *FlowJo*) was taken into account. The measurements above the histograms depict the portions of removed (red) and correct (green) signals within the example image. Scale bars = 50 µm.

Supplementary Figure 2

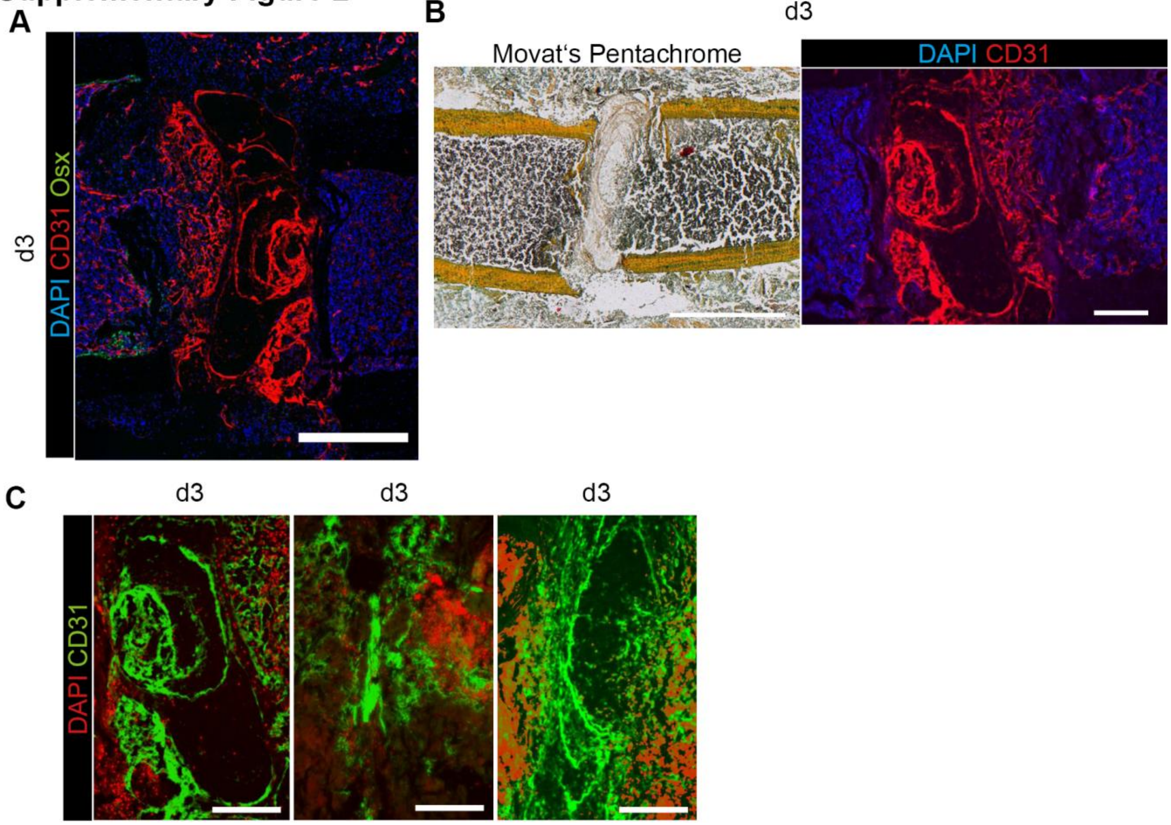

Supplementary Figure 2: Crosstalk between vessel formation and Osx^{+} osteoprogenitor cells during bone regeneration and accumulation of CD31 in the fracture hematoma at day 3

(A, B) At day 3 the residuals of the fracture hematoma are still visible in Movat's Pentachrome staining and strongly CD31^{+}. Scale bars = 500 µm. (C) Images exemplifies that the residuals of the fracture hematoma are cell-free. Scale bars = 200 µm.

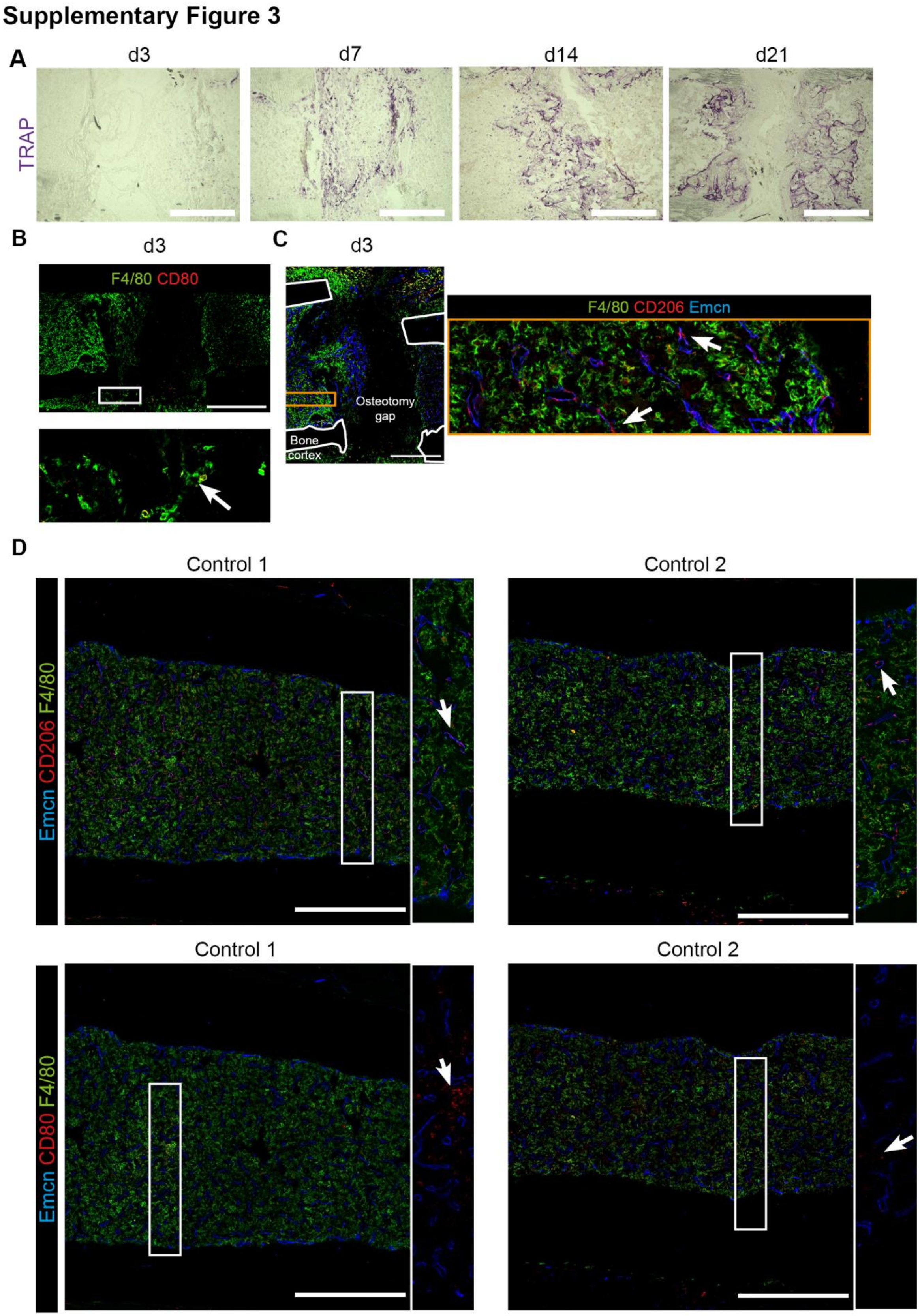

Supplementary Figure 3: Spatial localization of M1-like macrophages, CD206+ cells and scoring of polarization. (A) Exemplary images of TRAP staining within the fracture gap at day 3, 7,

14 and 21 show TRAP$^+$ cells in the fracture gap from day 7. (B) Immunofluorescence image of CD80 and F4/80 in an osteotomized bone 3 days post-surgery show few cells extramedular adjacent to the gap. Scale bar = 500 µm (C) Immunofluorescence image of CD206 and F4/80 show elongated F4/80$^-$ structures right next to endothelium suggesting CD206$^+$ endothelium during bone regeneration. (D) In contralateral control samples (n = 2) F4/80$^+$ cells are evenly distributed throughout the bone marrow. CD206$^+$F4/80$^+$ signals are detected along the endothelium of sinusoids. (E) CD80$^+$F4/80$^+$ cells were rare in control sections. No signal is detected extramedullar. Scale bars = 500 µm.

Supplementary Figure 4

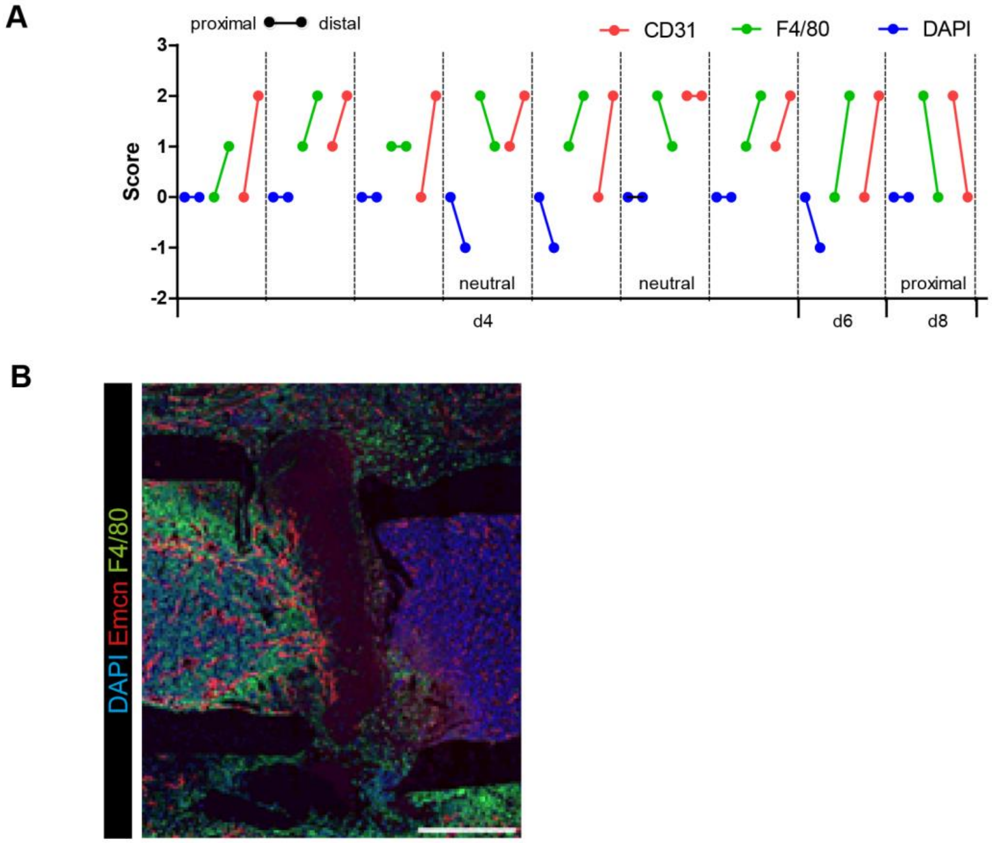

Supplementary Figure 4: Polarization scoring accumulated and individual scoring for the LIMB osteotomy-model show distal polarization. Polarization scoring for individual samples which were scored as described in the methods section. Samples were considered polarized when the scored values for F4/80 and vessel marker of proximal and distal resulted in a difference > 2 favoring one side (indicated: proximal, distal). Samples which scored a difference of < 2 were neutral (indicated: neutral). In total n = 20 samples were analyzed, 9 samples scored values other than 0 of which 2 are neutral, 1 polarized proximally and 6 polarized distally. (B) Polarization is also observed in the osteotomy-model. Scale bar = 500 µm.

Supplementary Figure 5

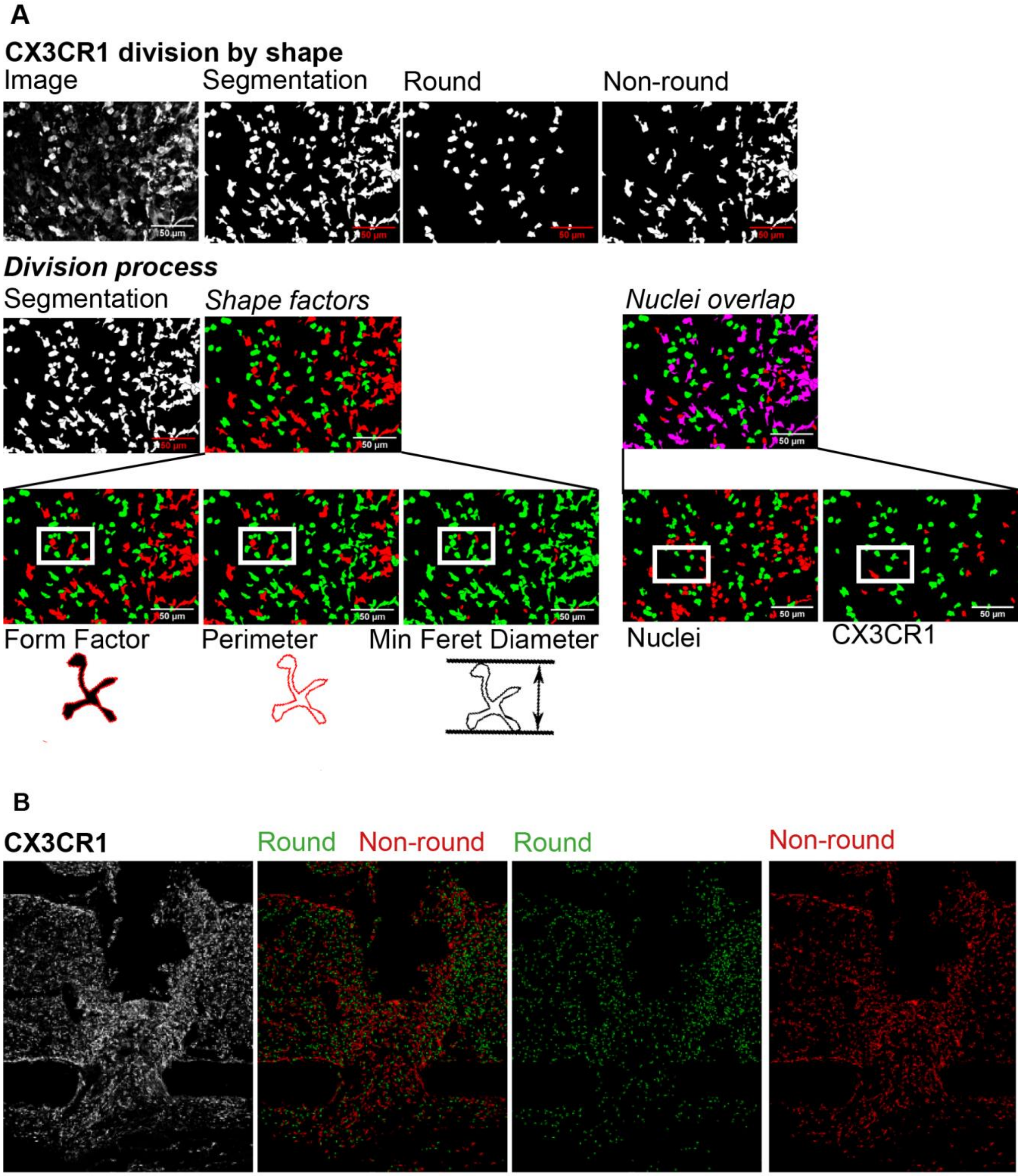

Supplementary Figure 5: Segmentation and division in round and non-round objects. (A) Exemplary segmentation process of a CX3CR1 fluororescent image, its segmentation via three shape factors (form factor, perimeter, and min Feret diameter). Objects were considered cells when they overlapped with a nucleus. (B) Exemplary result of the segmentation and division result.

Supplementary Figure 6

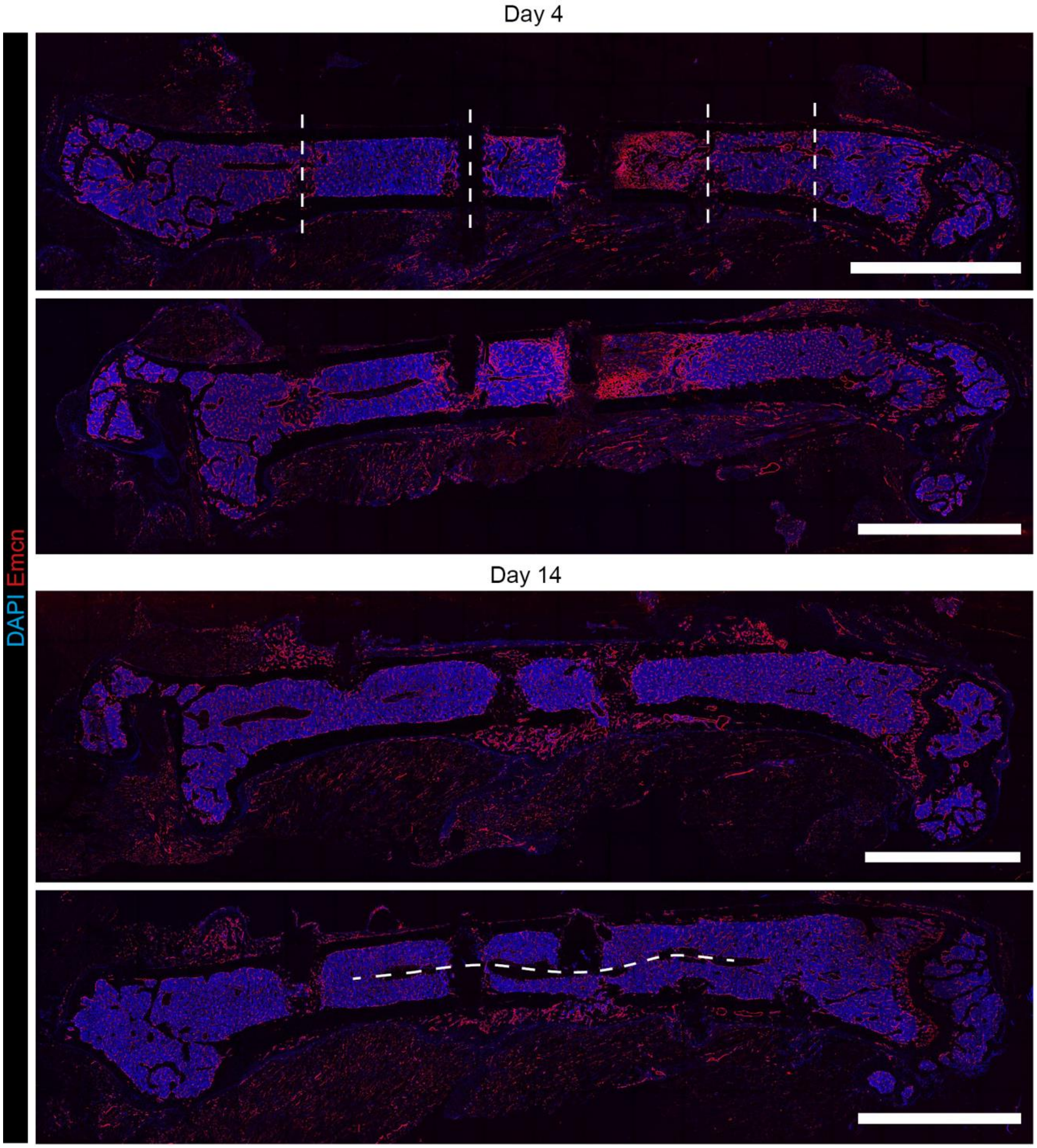

Supplementary Figure 6: Disrupted vascularization and polarization is transient. Exemplary immunofluorescence overview images of complete bones stained for Emcn in samples 4 and 14 days post-osteotomy show type H endothelium in damaged tissue and around the four screws (straight dashed lines). The main sinus is regenerated and fully visible at day 14. Scale bars = 2500 µm. n(day 4) = 3, n(day 14) = 2. Pictures were acquired using a 4x objective in tile scan mode at a Zeiss LSM880.

Supplementary Movie 1-4: Longitudinal intravital microendoscopy of the osteotomy gap using CX3CR1:GFP mice and Qtracker 655 (red). Low numbers of motile GFP^+ cells (green) enter the osteotomy gap in the field of view 2 days post-surgery and populate the observed volume before endothelium enters the volume, in close proximity to motile and sessile GFP^+ cells. The vascular network continues to remodel until day 6. Images 250 x 250 µm. Scale bar = 100 µm. (1) Day 2. (2) Day 3. (3) Day 4. (4) Day 5.
